# Is Choosing Wisely Wise for Lobular Carcinoma in Patients Over 70 Years of Age? A National Cancer Database Analysis of Sentinel Node Practice Patterns

**DOI:** 10.1245/s10434-023-13886-6

**Published:** 2023-07-25

**Authors:** Nicole H. Goldhaber, Thomas O’Keefe, Jessica Kang, Sasha Douglas, Sarah L. Blair

**Affiliations:** 1 Department of Surgery, University of California San Diego Health, La Jolla, CA USA; 2 School of Medicine, University of California San Diego, La Jolla, CA USA

## Abstract

**Background:**

Controversy continues in the treatment of breast cancer in women over 70 years of age. In 2016, the Society of Surgical Oncology recommended against routine use of sentinel lymph node biopsy (SLNBx) as part of the ‘Choosing Wisely Campaign’. This study examines the oncologic safety of avoidance of routine SLNBx in patients over 70 years of age with invasive lobular carcinoma (ILC).

**Methods:**

The National Cancer Database was used to identify women with invasive ductal carcinoma (IDC) and ILC diagnosed between 2012 and 2020. Clinical and pathological staging, axillary staging, surgery type, and lymph node positivity between patients with IDC or ILC were compared.

**Results:**

Among women with T1 tumors, 85,949 (79.6%) patients with IDC and 12,761 (81.5%) patients with ILC underwent SLNBx (*p* < 0.001). Among patients who underwent SLNBx, those with IDC were more likely to have positive nodes (*n* = 7535, 8.8%) than those with ILC (*n* = 1041, 8.2%; *p* = 0.02). During the time interval of interest, for both IDC and ILC patients, the rate of axillary lymph node dissection decreased and rates of SLNBx or no axillary staging increased. On multivariate analysis, ILC histology was associated with use of SLNBx, but without nodal positivity.

**Conclusion:**

A trend de-escalation of axillary staging was identified in this study, however the majority of patients meeting the ‘Choosing Wisely’ criteria are still undergoing SLNBx. No increased risk of nodal positivity was identified among patients with ILC, suggesting that surgeons can continue to choose wisely and limit the use of SLNBx in women over 70 years of age with T1 ILC tumors.

The management of women aged 70 years and older diagnosed with breast cancer remains controversial. Furthermore, there exists a paucity of literature informing the treatment of invasive lobular carcinoma (ILC) in this select group. In 2016, the Society of Surgical Oncology (SSO) recommended against the routine use of sentinel lymph node biopsy (SLNBx) for patients aged 70 years or older with clinically node-negative, hormone receptor (HR)-positive, human epidermal growth factor receptor 2 (HER2)-negative invasive breast cancer as part of the ‘Choosing Wisely Campaign’.^1^ Previous retrospective analyses have demonstrated that ILC is more likely to present with extensive disease that is underappreciated on preoperative imaging.^2,3^ Given that the size of ILC has been associated with an increased risk of sentinel lymph node metastasis,^4,5^ this underestimation in ILC size from imaging calls into question whether avoidance of routine SLNBx in patients with ILC over 70 years of age is oncologically safe and appropriate.

The Choosing Wisely guidelines are based on studies that included mostly invasive ductal carcinoma (IDC) rather than ILC patients, and treatment guidelines do not currently differentiate between these two disease processes.^6^ The Choosing Wisely recommendation to not routinely perform SLNBx in women over 70 years of age was made on the basis of the results of several randomized prospective trials.^7–9^ For example, Martelli et al. evaluated the long-term safety of no axillary surgery for patients over 70 years of age with operable breast cancer and clinically negative axillary nodes who received adjuvant endocrine therapy.^7^ This study found that axillary surgery did not increase overall survival breast cancer-specific survival over 5 years. The cumulative 15-year incidence of axillary disease was 5.8% for patients with T1 disease who had no axillary staging versus 3.7% for patients with T1 disease who underwent axillary lymph node dissection (ALND). Similarly, the IBCSG 10-93 trial assessed whether omitting axillary surgery in the elderly would translate to improved quality of life with equivalent disease-free survival and overall survival.^8^ A total of 473 women underwent surgical resection of breast cancer with planned adjuvant tamoxifen and were randomly assigned to receive or omit surgical axillary staging. At years of follow-up, there was no difference in disease-free survival or overall survival. Furthermore, CALGB 9343, a prospective randomized trial in which patients with T1 stage estrogen receptor (ER)-positive disease were randomized to lumpectomy with tamoxifen or lumpectomy with both adjuvant tamoxifen and radiation. In the subset of patients who received no axillary surgery or radiation, only 3% developed ipsilateral axillary recurrence compared with no recurrences in patients who received radiation without axillary staging. Given the low axillary recurrence rate, even among those who had omitted nodal surgery and radiotherapy, the authors concluded that SLNBx may be safely omitted in this population.^9^

Given that ILC is less prevalent and therefore underrepresented in large clinical trials compared with IDC, the primary aim of this analysis was to use a large national population-based registry to investigate the oncologic safety of de-escalation of axillary surgery in carefully selected patients aged 70 years or older with T1, HR-positive, HER2-negative ILC by comparing differences in positivity rates of SLNBx in ductal versus lobular carcinomas. The secondary aim was to explore the trends in axillary staging over time in this low-risk group. With this information, we can further assess whether the Choosing Wisely guidelines are applicable for this specific patient population.

## Methods

The present study was conducted using data from the National Cancer Database (NCDB), a nationwide, facility-based, comprehensive clinical surveillance resource oncology dataset that catalogs de-identified hospital-based patient data from Commission on Cancer (CoC)-accredited programs, including over 70% of newly diagnosed cancers.^10,11^ The database represents a joint effort between the American College of Surgeons and the American Cancer Society. The UCSD Human Research Protections Program (HRPP) Institutional Review Board (IRB) deferred the need for formal approval of this study due to the use of publicly available, de-identified data. Cases were sorted for breast as the primary site using codes C50.0–C50.9 from the International Classification of Diseases for Oncology, Third Edition (ICD-O-3). Our cohort included women diagnosed with ductal and lobular histologic cancer subtypes only, given these subtypes represent the most prevalent breast cancer subtypes. Male patients were excluded due to small numbers and to reflect the previously referenced randomized trials, which did not include men. Furthermore, patients with in situ behavior designation were excluded, as were those with incomplete staging data, diagnoses prior to 2012 (at which time the NCDB began including axillary staging details specific to SLNBx), prior breast cancer events, and hormonal status other than ER+/PR+/HER2−, ER+/PR−/HER2− or ER−/PR+/HER2−, as well as those who underwent axillary staging other than none or SLNBx or ALND (including those with unknown or unrecorded data in this variable) and those with incomplete clinical and pathological staging or demographic data.

For patients who met the inclusion criteria, univariate comparison analyses were performed using differences in proportions testing and Pearson’s Chi-square testing for categorical variables, and Student’s *t*-test for continuous variables between patients over and under the age of 70 years, as well as between patients with IDC versus ILC for the prevalence of T1 stage lesions. Furthermore, the incidence and positivity rates of SLNBx were examined in patients aged 70 years and older with pathologic stage T1 ER- or PR-positive, HER2-negative tumors comparing between patients with IDC or ILC. Demographic and cancer-related variables collected included age (note that the NCDB groups patients aged >90 years into one group, all listed as 90 years of age), race (grouped into White, Black or non-Black/non-White), insurance (grouped into Medicaid, Medicare, other government insurance, private insurance, or not insured), Charlson–Deyo comorbidity index score (with a score of 0 indicating no comorbid conditions recorded, noting that patients with a score of 0 could still have comorbidities if they are conditions that are not included in the mapping table), facility type (grouped into academic, community, comprehensive community, or integrated network), facility location (grouped into New England, Middle Atlantic, South Atlantic, East Central, West Central, Mountain, Pacific), HR status, and clinical and pathologic staging (T staging further subdivided into T1a, T1b, T1c, To). Our primary outcome variables were axillary staging surgery (grouped into none, SLNBx, or ALND) and positive lymph node status.

Multivariable logistic regression analyses were performed to identify factors that are associated with the use of SLNBx compared with no axillary staging, as well as factors associated with positive lymph node status. For both regression analyses, age, race, insurance, Charlson–Deyo, facility type, facility location, hormonal status and histology were included as covariates, and odds ratios were calculated for each. pT staging was also included in the regression comparing axillary staging.

The rates and incidences of axillary staging strategies (no axillary staging surgery, SLNBx only, or ALND) for patients in the study cohort with IDC or ILC were also compared over time. All statistical analyses were conducted using RStudio version 4.0.3, and for all analyses described, statistical significance was declared for *p* values < 0.05.

## Results

Within the NCDB breast dataset, 3,690,137 patients were identified, among whom 587,402 patients met the inclusion criteria, including 356,327 (86%) with IDC and 84,388 (14%) with ILC. Among patients diagnosed with ILC, 28,334 (34%) were at or over the age of 70 years at the time of diagnosis. By comparison, 146,687 (29%) patients diagnosed with IDC were at or over the age of 70 years. Clinicodemographic features for these patient populations are shown in Table 1.

**Table 1:** Clinicodemographic features of patients diagnosed with IDC or ILC, including subgroups of patients diagnosed at or over age 70 years with IDC or ILC

	All included (*n* = 587,402)	IDC pT1, Age 70 years or older (*n* = 107,931)	ILC pT1, Age 70 years or older (*n* = 15,656)	*P *value
Age	63 (median)	75 (median)	75 (median)	0.308
	54, 71 (IQR)	72, 79 (IQR)	72, 79 (IQR)	95%CI = − 0.04, 0.13
Race	White: 502,112 (85.5%)	White: 96,644 (89.5%)	White: 14,086 (90.0%)	< 0.001
	Black: 53,094 (9.0%)	Black: 7,305 (6.8%)	Black: 1,193 (7.6%)*	
	Non-black/Non-white: 32,196 (5.5%)	Non-black/Non-white: 3,982 (3.7%)	Non-black/Non-white: 377 (2.4%)*	
Insurance	Medicare: 250,530 (42.7%)	Medicare: 95,249 (88.2%)	Medicare: 13,922 (88.9%)*	0.053
	Private: 290,371 (49.4%)	Private: 10,582 (9.8%)	Private: 1,474 (9.4%)	
	Medicaid: 32,482 (5.5%)	Medicaid: 1,290 (1.2%)	Medicaid: 160 (1.0%)	
	Other government insurance: 6,304 (1.1%)	Other government insurance: 498 ( < 1%)	Other government insurance: 57 ( < 1%)	
	Not insured: 7,715 (1.3%)	Not insured: 312 ( < 1%)	Not insured: 43 ( < 1%)	
Charlson-Deyo	0: 483,132 (82.2%)	0: 82,611 (76.5%)	0: 12,303 (78.6%)*	< 0.001
	1: 76,661 (13.1%)	1: 17,443 (16.2%%)	1: 2,312 (14.8%)*	
	2: 18,090 (3.1%)	2: 4,928 (4.6%)	2: 675 (4.3%)	
	3: 9,519 (1.6%)	3: 2,949 (2.7%)	3: 366 (2.3%)*	
Facility type	Comprehensive community: 245,337 (41.8%)	Comprehensive community: 48,278 (44.7%)	Comprehensive community: 6,843 (43.7%)*	< 0.001
	Academic program: 171,126 (29.1%)	Academic program: 26,840 (24.9%)	Academic program: 4,208 (26.9%)*	
	Integrated network: 127,621 (21.7%)	Integrated network: 23,848 (22.1%)	Integrated network: 3,522 (22.5%)	
	Community: 43,318 (7.4%)	Community: 8,965 (8.3%)	Community: 1,083 (6.9%)*	
Facility location	East Central: 133,915 (22.8%)	East Central: 25,300 (23.4%)	East Central: 3,550 (22.7%)*	< 0.001
	South Atlantic: 129,455 (22.0%)	South Atlantic: 24,604 (22.8%)	South Atlantic: 3,543 (22.6%)	
	West Central: 91,585 (15.6%)	West Central: 16,576 (15.4%)	West Central: 2,256 (14.4%)*	
	Middle Atlantic: 87,132 (14.8%)	Middle Atlantic: 15,865 (14.7%)	Middle Atlantic: 2,567 (16.4%)*	
	Pacific: 80,914 (13.8%)	Pacific: 14,024 (13.0%)	Pacific: 1,934 (12.4%)*	
	New England: 35,118 (6.0%)	New England: 6,481 (6.0%)	New England: 1,066 (6.8%)*	
	Mountain: 29,283 (5.0%)	Mountain: 5,081 (4.7%)	Mountain: 740 (4.7%)	
Hormonal status	ER+PR+HER2-: 521,304 (88.7%)	ER+PR+HER2-: 96,889 (89.8%)	ER+PR+HER2-: 13,158 (84.0%)*	< 0.001
	ER+PR-HER2-: 61,265 (10.4%)	ER+PR-HER2-: 10,388 (9.6%)	ER+PR-HER2-: 2,480 (15.8%)*	
	ER-PR+HER2-: 4,833 ( < 1.0%)	ER-PR+HER2-: 654 ( < 1.0%)	ER-PR+HER2-: 18 ( < 1.0%)*	
Clinical T-stage	cT1a: 41,384 (7.0%)	cT1a: 11,376 (10.5%)	cT1a: 1,199 (7.7%)*	< 0.001
	cT1b: 134,386 (22.9%)	cT1b: 36,104 (33.5%)	cT1b: 4,877 (31.2%)*	
	cT1c: 176,361 (30.0%)	cT1c: 34,722 (32.2%)	cT1c: 5,715 (36.5%)*	
	cTo: 235,271 (40.1%)	cTo: 25,729 (13.8%)	cTo: 3,865 (24.7%)*	
Pathologic T-stage	pT1a: 54,058 (9.2%)	pT1a: 14,445 (13.4%)	pT1a: 1,192 (7.6%)*	< 0.001
	pT1b: 135,792 (23.1%)	pT1b: 38,093 (35.3%)	pT1b: 4,331 (27.7%)*	
	pT1c: 220,975 (37.6%)	pT1c: 53,833 (49.9%)	pT1c: 9,944 (63.5%)*	
	pTo: 176,577 (30.1%)	pTo: 1,560 (1.4%)	pTo: 189 (1.2%)*	
Axillary staging	Sentinel Lymph Node Biopsy: 479,825 (81.6%)	Sentinel Lymph Node Biopsy: 85,949 (79.6%)	Sentinel Lymph Node Biopsy: 12,761 (81.5%)*	< 0.001
	None: 47,667 (8.1%)	None: 17,150 (15.9%)	None: 2,216 (14.2%)*	
	Axillary dissection: 59 ,910 (10.2%)	Axillary dissection: 4 ,832 (4.5%)	Axillary dissection: 679 (4.3%)	

* Indicates statistical significance between headings within categorical variables using differences in proportions. *IDC* Invasive ductal carcinoma, *ILC* Invasive lobular carcinoma, *IQR* Interquartile range, *CI* Confidence interval, *ER* Estrogen receptor, *PR* Progesterone receptor, *HER2* Human epidermal growth factor receptor 2, *T* Tumor, *c* Clinical, *p* Pathologic, *a/b/c* Tumor substage, *o* Other (not T1a/T1b/T1c), *+* indicates positive expression, *−* indicates negative expression

For patients over 70 years of age with pT1 breast cancer, race, Charlson–Deyo comorbidity score, facility type, facility location, hormonal status, and both clinical and pathologic T staging were all significantly different between patients with IDC versus ILC lesions. Most notably, patients in this cohort with ILC had a greater proportion of Medicare insurance (88.9% vs. 88.2%; *p* = 0.01) and a lower proportion of high (three) Charlson–Deyo comorbidity scores (2.3% vs. 2.7%; *p* < 0.01). Furthermore, patients with IDC were more likely to be ER- and PR-positive compared with patients with ILC (89.8 vs. 84%; *p* < 0.01) and less likely to have ER-positive and PR-negative disease (9.6 vs. 15.8%; *p* < 0.01).

**Fig. 1:**
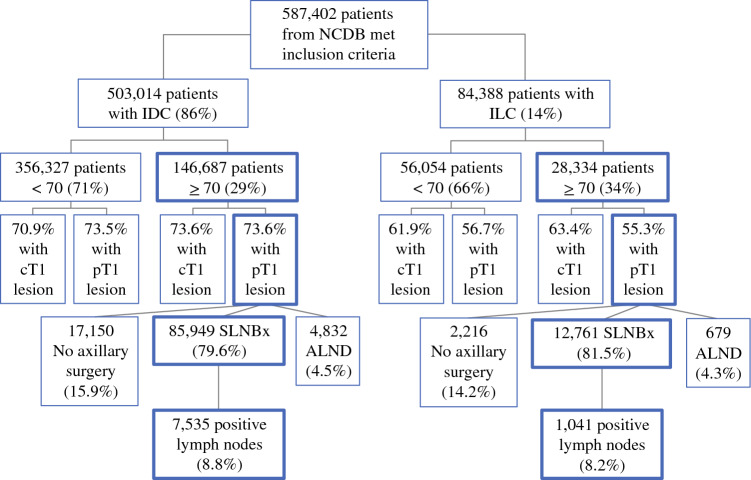
Patient cohort from the NCDB comparing patients diagnosed with IDC or ILC, by clinical and pathologic T stage and axillary staging surgery type, as well nodal positivity. *ALND* axillary lymph node dissection, *SLNBx* sentinel lymph node biopsy, *NCDB* National Cancer Database, *IDC* invasive ductal carcinoma, *ILC* invasive lobular carcinoma

Among patients with ILC, those 70 years of age and older were more likely to have clinical stage T1 (cT1) lesions compared with those under 70 years of age (63.4 vs. 51.9%; *p* < 0.01); however, they were less likely to have pathological stage T1 (pT1) lesions (55.3 vs. 56.7%; *p* < 0.01). In comparison, patients with IDC aged 70 years and older were more likely to have cT1 lesions (73.6 vs. 70.9%; *p* < 0.01) and equally likely to have pT1 lesions (73.6 vs. 73.5%; *p* = 0.35) compared with those under 70 years of age. It is important to note that percentage differences in all cases listed are quite small and are likely not clinically significant (Fig. 1).

In women aged 70 years or older with pT1 tumors, 85,949 (79.6%) of patients with IDC and 12,761 (81.5%) of patients with ILC underwent SLNBx (*p* < 0.01). Among those patients who underwent SLNBx, those with IDC were more likely to have positive lymph nodes (*n* = 7535, 8.8%) compared with those with ILC (*n* = 1041, 8.2%; *p* = 0.02) [Fig. 1].

**Fig. 2:**
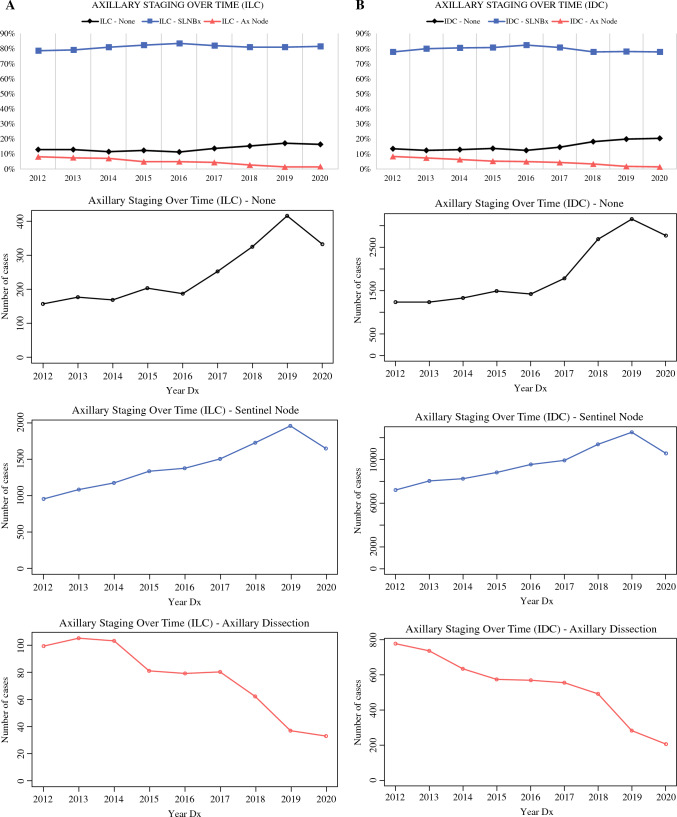
Trends in axillary staging over time for patients with IDC or ILC. **A** Percentage of axillary staging surgery type or no axillary staging, by year of patient diagnosis. **B** Number of cases of each surgery type or no axillary staging, by year of patient diagnosis. *SLNBx* sentinel lymph node biopsy, *IDC* invasive ductal carcinoma, *ILC* invasive lobular carcinoma

Rates of each axillary staging strategy were compared for women aged 70 years or older with stage T1 disease, for each year in the time frame of interest (from 2012 to 2020), for patients with ILC and IDC (Fig. 2). In both IDC and ILC patients, the rate and incidence of ALND decreased, while the rates and incidences of SLNBx and no axillary staging increased over time. Interestingly, there was a drop in all axillary staging methods from 2019 to 2020, possibly as a result of the decrease in case numbers during the coronavirus disease 2019 (COVID-19) pandemic.^16–18^

The results of multivariable logistic regression analyses for the utilization of SLNBx and lymph node positivity are summarized in Table 2. Most notably, utilization of SLNBx was associated with younger age (among the cohort all aged over 70 years), White race, Medicare or private insurance, a score of zero on the Charlson–Deyo score, stage pT1b and pT1c lesions, and ILC histology (interestingly, facilities in New England are less likely to get sentinel node). Nodal positivity was associated with younger age (among the cohort all aged over 70 years), Black race, a score of 1 on the Charlson–Deyo score, and West Central facility location (again with New England less likely to have nodal positivity, this time with Medicare and private insurance). Although ILC patients were more likely to undergo SLNBx in T1 tumors, they were not more likely to be positive.

**Table 2:** Multivariate analysis examining the likelihood of undergoing sentinel node biopsy and the factors predicting node positivity in patients who underwent sentinel node biopsy

Variable	Odds ratio		Odds ratio	
	Sentinel node biopsy	*P *value	Positive lymph nodes	*P *value
Age	0.876	< 0.001	0.981	< 0.001
Race	ref	ref	ref	ref
Black	1.029	0.555	0.724	< 0.001
Other	1.184	< 0.001	0.74	< 0.001
White				
Insurance	ref	ref	ref	ref
Medicaid	1.176	0.016	0.735	< 0.001
Medicare	0.783	0.094	1.115	0.535
Not Insured	1.123	0.37	0.877	0.409
Other government	1.126	0.095	0.763	0.002
Private				
Charlson-Deyo	ref	ref	ref	ref
0	0.887	< 0.001	1.097	< 0.001
1	0.713	< 0.001	1.031	0.528
2	0.676	< 0.001	1.006	0.924
3				
Facility type	ref	ref	ref	ref
Academic/Research	1.34	< 0.001	1.077	0.061
Community	1.369	< 0.001	0.969	0.219
Comprehensive community	1.401	< 0.001	0.963	0.202
Integrated network				
Facility location	ref	ref	ref	ref
East central	1.121	< 0.001	0.954	0.167
Middle Atlantic	1.03	0.438	1.142	0.007
Mountain	0.55	< 0.001	0.784	< 0.001
New England	1.158	< 0.001	1.02	0.574
Pacific	1.425	< 0.001	1.088	0.004
South Atlantic	1.491	< 0.001	1.148	< 0.001
West Central				
Hormonal status	ref	ref	ref	ref
ER-PR+HER2-	0.712	0.002	0.982	0.894
ER+PR-HER2-	0.697	0.001	1.033	0.807
ER+PR+HER2-				
Histology	ref	ref	ref	ref
IDC	1.15	< 0.001	0.957	0.143
ILC				

*IDC* Invasive ductal carcinoma, *ILC* invasive lobular carcinoma, *ER* Estrogen receptor, *PR* Progesterone receptor, *HER2* Human epidermal growth factor receptor, *ref* Reference, *T* Tumor, *p* Pathologic, *a/b/c* Tumor substage, *o* Other (not T1a/T1b/T1c), + indicates positive expression, − indicates negative expression

## Discussion

IDC is by far the most common type of breast cancer, with approximately 80% of breast cancers being ductal in nature and only 5–15% being lobular in nature^19,20^; thus, ILC is underrepresented in clinical trials for breast cancer overall compared with IDC. ILC is more common in patients above the age of 50 years compared with patients under 50 years of age, and the incidence of ILC in this population is rising.^19^ On clinical presentation, patients with ILC tend to present with a mass or a vague, poorly circumscribed nodularity.^5^ The indolent and infiltrative growth pattern of ILC tumors make early diagnosis difficult with mammography. Furthermore, because of their low cellularity, these cancers can be easily missed on fine needle aspiration or needle core biopsy. For these reasons, ILC lesions tend to be diagnosed later than IDC lesions and are often clinically understaged.^2^

For example, Molland et al. compared 182 patients with ILC versus 1612 patients with IDC and found that patients with ILC presented with significantly larger tumors, were more likely to require re-excision for positive margins or require mastectomy, but the rate of positive axillary nodes was similar.^21^ Additionally, these results were confirmed by a large population-based study from The Netherlands.^22^ From the present analysis, it also appears that clinical T1 lesions are less likely to remain pathologic T1 for ILC in women over 70 years of age versus IDC; this may explain, at least in part, why surgeons are more likely to perform an SLNBx for ILC cT1 lesions than for IDC cT1 lesions. Furthermore, this brings into question whether it is safe to de-escalate axillary staging if pathologic T stage may be larger on pathology. Breast MRI has been shown to better correlate with extent of disease among patients with ILC, allowing surgeons to more accurately identify appropriate patients with T1 tumors for whom more aggressive surgery and axillary staging can be safely avoided.^23^

The American Board of Internal Medicine (ABIM) Foundation launched a national initiative called ‘Choosing Wisely’ to prompt provider discussions about the appropriate use of tests, treatments, and procedures based on evidence-driven medicine.^24^ In conjunction with the SSO in 2016, five recommendations were released. The first recommendation stated, “Don’t routinely use sentinel node biopsy in clinically node-negative women ≥70 years of age with early-stage HR-positive, HER2-negative invasive breast cancer”.^1^ This recommendation was based on several prospective trials highlighting that SLNB had no impact on locoregional recurrence or breast cancer-specific mortality in this group of patients.^6–9^

Welsh et al. developed a model to aid in predicting positive SLNBx in women over 70 years of age, based on data from NCDB.^25^ They found that patients with ILC were more likely to undergo axillary surgery compared with patients with IDC, while those with invasive mucinous, tubular, or papillary carcinoma were less likely. They also found that the lowest risk for nodal positivity was patients with grade 1, clinical T1mi-T1c (≤2.0 cm), or grade 2, clinical T1mi-T1b (≤1.0 cm) tumors, with a positive node rate of 7.8% (95% confidence interval [CI] 7.4–8.3%). Patients not in the low-risk group (which included all grade 3 tumors, cT2+ tumors, and grade 2, clinical T1c tumors) had a positive node rate of 22.3% (95% CI 21.7–22.8%), with a relative risk of nodal positivity of 2.84 (95% CI 2.68–3.02; *p* < 0.001). On multivariate analysis, Welsh et al. found an increased risk of node positivity in patients with ILC (1.18 [1.09–1.28]). In the present analysis, focusing on T1 tumors, we found ILC had a slightly lower risk of node positivity in patients who underwent SLNBx. In the study by Welsh et al., 11% of patients did not undergo axillary staging surgery.^25^ The Choosing Wisely campaign was published in 2016, while the study by Welsh et al. was published in 2017. The present analysis includes NCDB data from 2012 to 2020. We found the omission of axillary staging in the defined cohort of interest increased to about 20% even by the terminal years of the study, which suggests that further progress could be made to improve compliance with the Choosing Wisely campaign guidelines.

Carleton et al. performed a single-institutional retrospective review of a prospectively collected database including 145 patients with ILC and 971 patients with IDC, all with stage 1 ER-positive tumors.^26^ Among patients who underwent SLNBx, there was no difference in the lymph node positivity rate between IDC and ILC. Similarly, there was no difference in the axillary recurrence rate. In the present study utilizing the NCDB, we analyzed data from thousands of patients across many centers, giving population-based data over a longer period. A major limitation of NCDB is a lack of local and regional recurrence data: however, the current study demonstrates there was a low rate of positive axillary lymph nodes in this carefully selected cohort of patients including women over 70 years of age with pT1 stage ER- and/or PR-positive HER2-negative tumors independent of ductal versus lobular histology.

However, there are limitations to our study. The NCDB only captures patients treated at CoC-accredited hospitals, and inherent to any large national registry-based study, there may be errors in abstraction and coding. This study is subject to the same limitations as prior retrospective reports, such as selection bias and incomplete or incorrect variables. In addition, there are variables that are not presently coded within the database, including, but not limited to, family history, prior hormone therapy exposure, prior negative breast biopsy results, life expectancy, and functional status, which all contribute significantly to breast cancer risk and may inform decision making regarding axillary staging choice. Additionally, this analysis focuses on practice trends and not on the impact of breast surgery on oncologic and patient-reported outcomes.

It is also important to note that there have been several changes in the data collection and descriptions of variables in the NCDB over time. Specifically, with regard to staging, the AJCC clinical and pathologic stage groups included in the NCDB breast dataset are a TNM-based system coded or reported according to the edition corresponding to the patient’s diagnosis year. The 5th Edition of the American Joint Committee on Cancer (AJCC) staging manual is used to represent patients’ cases diagnosed from 1998 through 2002, while the 6th Edition describes the anatomic extent of disease for patients diagnosed from 2003 through 2009. Patients diagnosed in 2010–2017 are staged according to the 7th Edition of the AJCC staging manual data, whereas for cases diagnosed in 2018 and later, the AJCC 8th Edition is used.^12,13^ As a result, caution was taken when using staging information due to the shifts in definitions over time.

In addition, it is also important to note that due to related changes in staging and reporting, cancer grade was not included in this analysis. In 2018, this item was not only transitioned to a Site-Specific Data Items (SSDI) reporting item as described for hormonal status, but additional changes were made regarding recommendations for grade reporting to shift from well/moderate/poorly/undifferentiated to low/intermediate/high Nottingham combined histologic grade (Nottingham modification of the Scarff-Bloom-Richardson [SBR] score grading system).^14,15^ Finally, over time the NCDB has documented sentinel node involvement differently; we attempted to minimize this influence by starting our analysis at 2012 when the database coded for sentinel node procedures specifically.

Despite these limitations of the NCDB dataset, we did have a large sample size of patients who met our inclusion criteria. As a result, we discovered many relationships between variables that were technically statistically significant results. However, due to the power of large numbers in NCDB, we do believe that we detected statistically significant differences (for example, with regard to several demographic variables, including race, insurance status, geographic location, and even HR status) that may not be as clinically significant.

## Conclusion

Although there is a trend towards less axillary staging in this highly selected low-risk group of women over 70 years of age with T1 ILC tumors, there remains significant room for improvement. Together with the lack of increased nodal positivity in patients over 70 years of age with pT1 ILC lesions compared with IDC lesions, our results suggest we should be reassured that surgeons can continue to ‘choose wisely’ and limit the use of SLNBx in women over 70 years of age with ILC T1 tumors, when deemed appropriate. These results can help providers guide the management of ILC in women over age 70 years.
